# Biogeographical Patterns of Bacterial Communities and Their Antibiotic Resistomes in the Inland Waters of Southeast China

**DOI:** 10.1128/spectrum.00406-22

**Published:** 2022-06-23

**Authors:** Peiju Fang, Peng Xiao, Fengjiao Tan, Yuanyuan Mo, Huihuang Chen, Uli Klümper, Thomas U. Berendonk, Jun Yang

**Affiliations:** a Aquatic EcoHealth Group, Fujian Key Laboratory of Watershed Ecology, Ningbo Observation and Research Station, Key Laboratory of Urban Environment and Health, Institute of Urban Environment, Chinese Academy of Sciences, Xiamen, China; b University of Chinese Academy of Sciences, Beijing, China; c Institute of Hydrobiology, Technical University of Dresden, Dresden, Germany; d Zhejiang Key Laboratory of Urban Environmental Processes and Pollution Control, CAS Haixi Industrial Technology Innovation Center in Beilun, Ningbo, China; University of California Davis

**Keywords:** antibiotic resistance genes, bacterioplankton, geographical distribution, high-throughput quantitative PCR, high-throughput sequencing

## Abstract

Freshwater ecosystems are important sources of drinking water and provide natural settings for the proliferation and dissemination of bacteria and antibiotic resistance genes (ARGs). However, the biogeographical patterns of ARGs in natural freshwaters and their relationships with the bacterial community at large scales are largely understudied. This is of specific importance because data on ARGs in environments with low anthropogenic impact is still very limited. We characterized the biogeographical patterns of bacterial communities and their ARG profiles in 24 reservoirs across southeast China using 16S rRNA gene high-throughput sequencing and high-throughput-quantitative PCR, respectively. We found that the composition of both bacterial communities and ARG profiles exhibited a significant distance-decay pattern. However, ARG profiles displayed larger differences among different water bodies than bacterial communities, and the relationship between bacterial communities and ARG profiles was weak. The biogeographical patterns of bacterial communities were simultaneously driven by stochastic and deterministic processes, while ARG profiles were not explained by stochastic processes, indicating a decoupling of bacterial community composition and ARG profiles in inland waters under relatively low-human-impact at a large scale. Overall, this study provides an overview of the biogeographical patterns and driving mechanisms of bacterial community and ARG profiles and could offer guidance and reference for the control of ARGs in drinking water sources.

**IMPORTANCE** Antibiotic resistance has been a serious global threat to environmental and human health. The “One Health” concept further emphasizes the importance of monitoring the large-scale dissemination of ARGs. However, knowledge about the geographical patterns and driving mechanisms of bacterial communities and ARGs in natural freshwater environments is limited. This study uncovered the distinct biogeographical patterns of bacterial communities and ARG profiles in inland waters of southeast China under low-anthropogenic impact at a large scale. This study improved our understanding of ARG distribution in inland waters with emphasis on drinking water supply reservoirs, therefore providing the much-needed baseline information for future monitoring and risk assessment of ARGs in drinking water resources.

## INTRODUCTION

Antibiotic-resistant bacteria (ARB) and antibiotic resistance genes (ARGs) are ancient and common in the environment (1). However, human activities have accelerated their evolution, proliferation, and dissemination in and across different ecosystems (2, 3). ARGs can be transferred between environmental bacteria and human pathogens by horizontal gene transfer through mobile genetic elements (MGEs) (4). Recently, both ARB and ARGs have been regarded as emerging pollutants because they can threaten environmental and human health worldwide (5, 6). More recently, numerous studies have reported abundant ARGs in wastewater, sediment, soil, and air environments (7–11). Pollution with multiresistant ARB and mobile ARGs that are coupled with additional resistance to various pollutants (including antibiotic residues, biocides, and heavy metals) on the same MGE has made aquatic ecosystems crucial hot spots for the acquisition and dissemination of both ARB and ARGs in human-dominated regions (12–14).

Aquatic ecosystems provide a range of ecosystem services, including food, water, and energy, which are closely related to human survival, development, and health (15–17). However, freshwater bodies often serve as both receiving wastewater effluents and drinking water sources. They may hence harbor diverse ARGs which might in turn be transferred to pathogenic bacteria (18, 19). Currently, an increasing number of studies have documented the global distribution of ARGs in wastewater (4, 20), but little is known about the geographical patterns and driving mechanisms of ARGs in natural freshwater environments at regional and continental scales (3, 21). Recently, the “One Health” concept, which was suggested as an important perspective to address problems associated with antibiotic resistance, emphasizes the necessity of monitoring the large-scale dissemination of ARGs locally, regionally, globally, and across human, animal, and environmental spheres (22). Hence, monitoring and assessment of the distribution, spread, and drivers of ARG diversity and abundance in inland waters on a large scale could provide a reference for designing policy and intervention strategies to combat antibiotic resistance.

Previous studies have demonstrated that the spatial distribution of freshwater microbial communities exhibited a distance-decay pattern, which means that the community similarity between samples decreases with increasing geographical distance (23). Their taxonomic composition differs across different geographical regions. Further, certain functional trait profiles exhibited a consistent pattern where taxonomic composition correlated with geographical variables (24), while other studies reported that the distribution of bacterial taxonomy and function is governed by different processes and mechanisms (25, 26). However, very few studies have explored biogeographical patterns of bacterial taxonomic community composition in connection with ARG profiles in the environments under relatively low anthropogenic impact such as natural freshwater (3).

In this study, we investigated the distribution patterns of bacterial taxonomic community composition and ARG profiles in inland waters of southeast China along a distance gradient of 1800 km (Fig. S1). We characterized the bacterial communities and ARG profiles in 24 reservoirs (Table S1) using high-throughput sequencing and high-throughput quantitative PCR (HT-qPCR), respectively. Nineteen of these reservoirs were important drinking water sources for residents. The aims of this study were to (i) compare the biogeographical patterns of bacterial communities and ARG profiles; (ii) elucidate the driving mechanisms and processes underlying these biogeographical patterns; (iii) determine the relationship between spatial distribution patterns between bacterial communities and ARG profiles. Our study could provide a reference baseline on ARG abundance at a large scale and, hence, guidance for future risk assessment and control strategies for ARGs in inland natural waters.

## RESULTS

### Biogeographical pattern of ARG profiles and bacterial communities.

**FIG 1:**
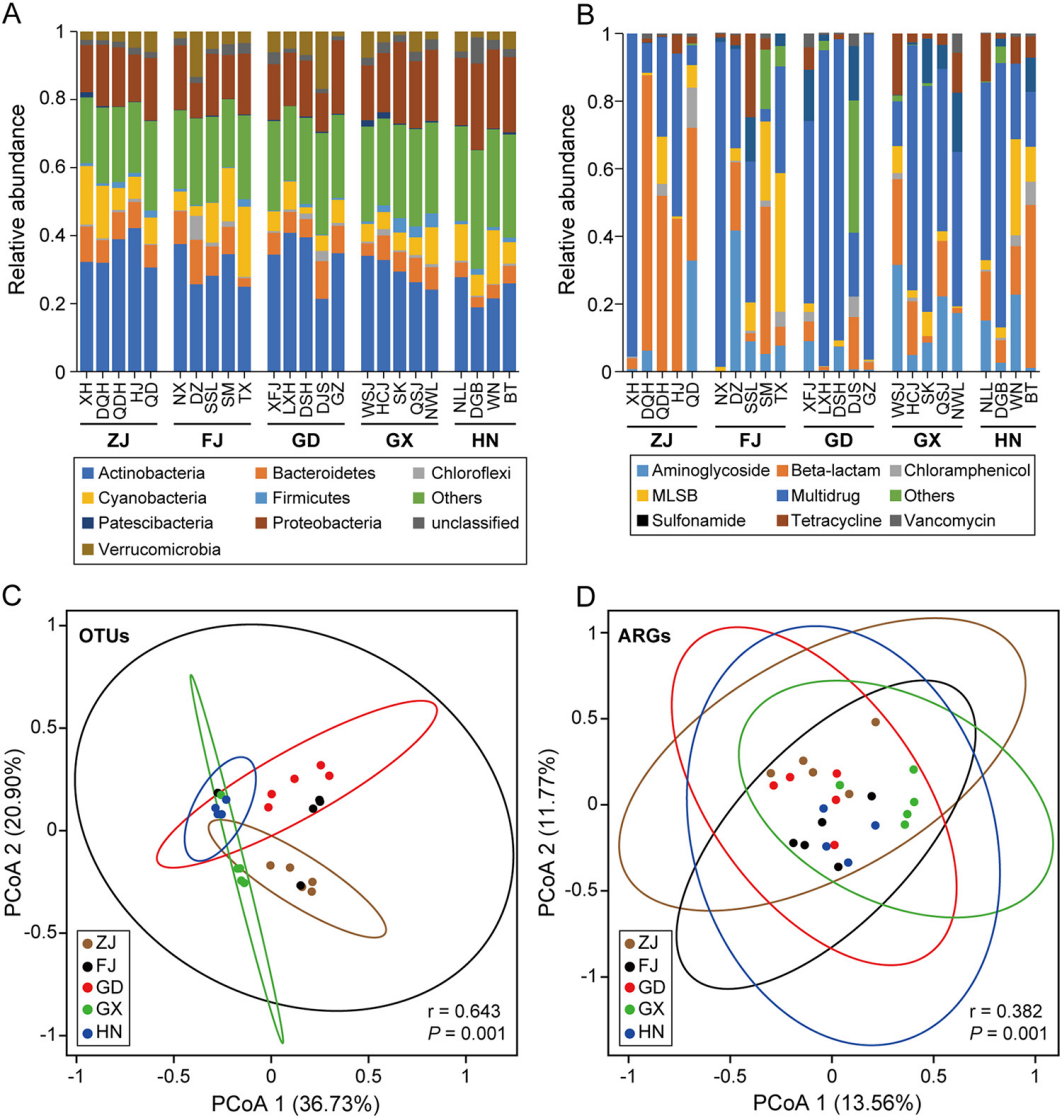
Composition of bacterial communities and ARG profiles in 24 reservoirs, southeast China. Relative abundances of (A) bacteria at the phylum level (Others, bacterial phyla at relative abundance below 3%) and (B) ARGs at the class level. The dominant phyla were Actinobacteria (dark blue), Cyanobacteria (yellow), and Proteobacteria (brown). The dominant ARG classes were beta-lactams (orange), MLSB (yellow), and multidrug (dark blue). Principal coordinates analysis (PCoA) of (C) bacterial communities and (D) ARG profiles based on Bray-Curtis similarity calculated at OTU and gene-level showing their distribution patterns. Statistics r and *P* values were calculated by analysis of similarity (ANOSIM), higher r values indicate a stronger difference between groups. Ellipses represent 95% confidence intervals of group distributions. ZJ: Zhejiang province; FJ: Fujian province; GD: Guangdong province; GX: Guangxi province; HN: Hainan province.

**FIG 2:**
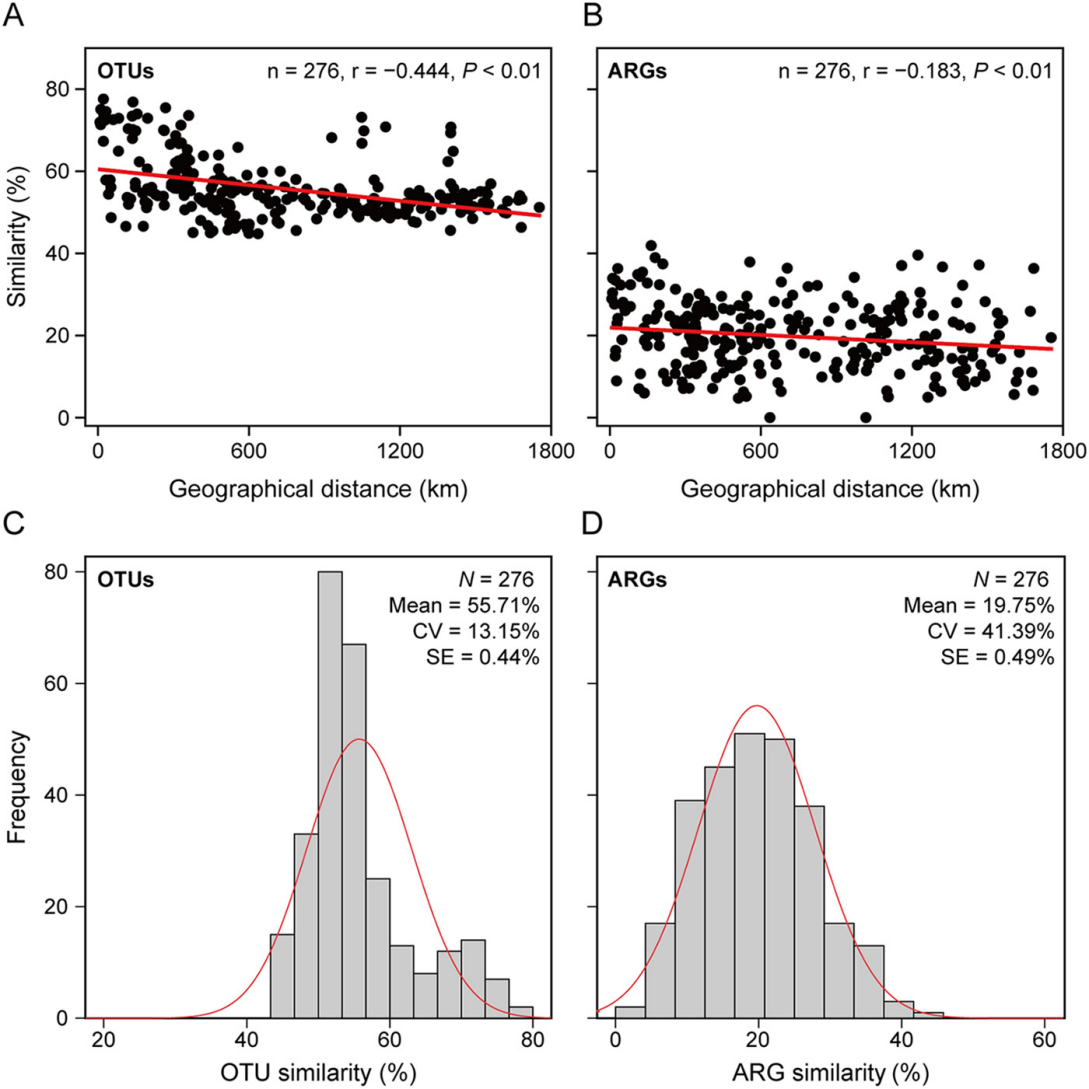
Bray-Curtis similarities of bacterial communities and ARG profiles. Spearman’s rank correlation between geographical distance and similarity of (A) bacterial communities, or (B) ARG profiles. The frequency distributions of Bray-Curtis similarities of bacterial communities (C) and ARG profiles (D). CV indicates the variation coefficient of the similarity, and SE indicates standard error.

Different biogeographical patterns of bacterial communities and ARG profiles were identified based on Bray-Curtis similarity. The dominant phyla across all the 24 reservoirs were Actinobacteria, Proteobacteria, and Cyanobacteria (Fig. 1A). In contrast, the composition of ARG profiles showed a remarkable variation between water bodies. Multidrug resistance genes were the main ARGs identified in most reservoirs, but some water bodies were dominated by aminoglycoside, beta-lactam, and macrolide-lincosamide-streptogramin B (MLSB) resistance genes (Fig. 1B). Principal coordinate analysis (PCoA) analysis indicated different distribution patterns between bacterial communities (Fig. 1C) and ARG profiles (Fig. 1D). The difference in bacterial community compositions among five provinces (groups) was higher than that of ARG profiles (Global R: 0.643 for OTUs and 0.382 for ARGs), indicating a stronger geographical distribution pattern of the bacterial community. Further, the Bray-Curtis similarity of bacterial communities was significantly and negatively correlated with geographical distance (r = −0.444, *P* < 0.01). Still, the ARGs-distance relationship was much weaker than that observed for bacterial community composition (r = −0.183, *P* < 0.01), suggesting that bacterial communities exhibited a stronger distance-decay pattern than ARG profiles (Fig. 2A and B). Further, the similarity of bacterial communities was higher than that of ARG profiles, as depicted by the frequency distribution of Bray-Curtis similarity (Fig. 2C and D). Taken together, these results indicate distinct biogeographical patterns of bacterial communities and ARG profiles and demonstrate that ARG profiles are more variable than bacterial community compositions. Thus, the biogeographical distribution patterns of bacterial taxonomic composition were decoupled from antibiotic resistance traits in these freshwater water bodies.

Across the 24 sampled reservoirs, a total of 165 (out of 285 tested) unique ARGs and 8 (out of 10 tested) MGEs were successfully amplified in at least one sample. Unsurprisingly, *cIntI* (a clinically associated class 1 integron-integrase gene) was not detected in any of the freshwater reservoirs perhaps indicating a low anthropogenic impact on these water bodies (Fig. S2). The absolute abundance of ARGs ranged from 2.22 × 10^6^ to 4.47 × 10^8^ copies/liter, while the absolute abundance of MGEs ranged from 3.56 × 10^5^ to 2.61 × 10^8^ copies/liter (Table S2). ARGs were detected in all samples, whereas MGEs were not detected in the Shanmei and Qingshuijiang reservoirs (Fig. S2). Although the samples in this study were taken covering a large latitudinal distance, the absolute, as well as the relative abundance of total ARGs and MGEs, were not significantly correlated with latitude. However, the richness of ARGs significantly decreased with increasing latitude (r = −0.615, *P* < 0.001) (Fig. S3). Further, these genes differed significantly among different water bodies. The most prevalent gene (*sul2*) occurred in 15 water bodies (Fig. S4), while 53 genes were detected only at a single sample site. Few ARGs were shared among provinces or even among water bodies in the same province, while more unique genes were detected in these samples (Fig. S5).

Regarding community diversity, 4971 unique bacterial operational taxonomic units (OTUs) across the 24 sites were obtained with the bacterial richness varying between 1908 and 3114 OTUs across the 24 sampling water bodies. The absolute abundance of the 16S rRNA gene ranged from 6.07 × 10^8^ to 1.01 × 10^10^ copies/liter, while the Shannon-Wiener index of bacterial communities varied from 4.41 to 5.95 (Fig. S3). However, the abundance, richness, and Shannon-Wiener index of bacterial communities did not show any significant relationship with latitude (Fig. S3).

### Assembly processes of ARG profiles and bacterial communities.

**FIG 3:**
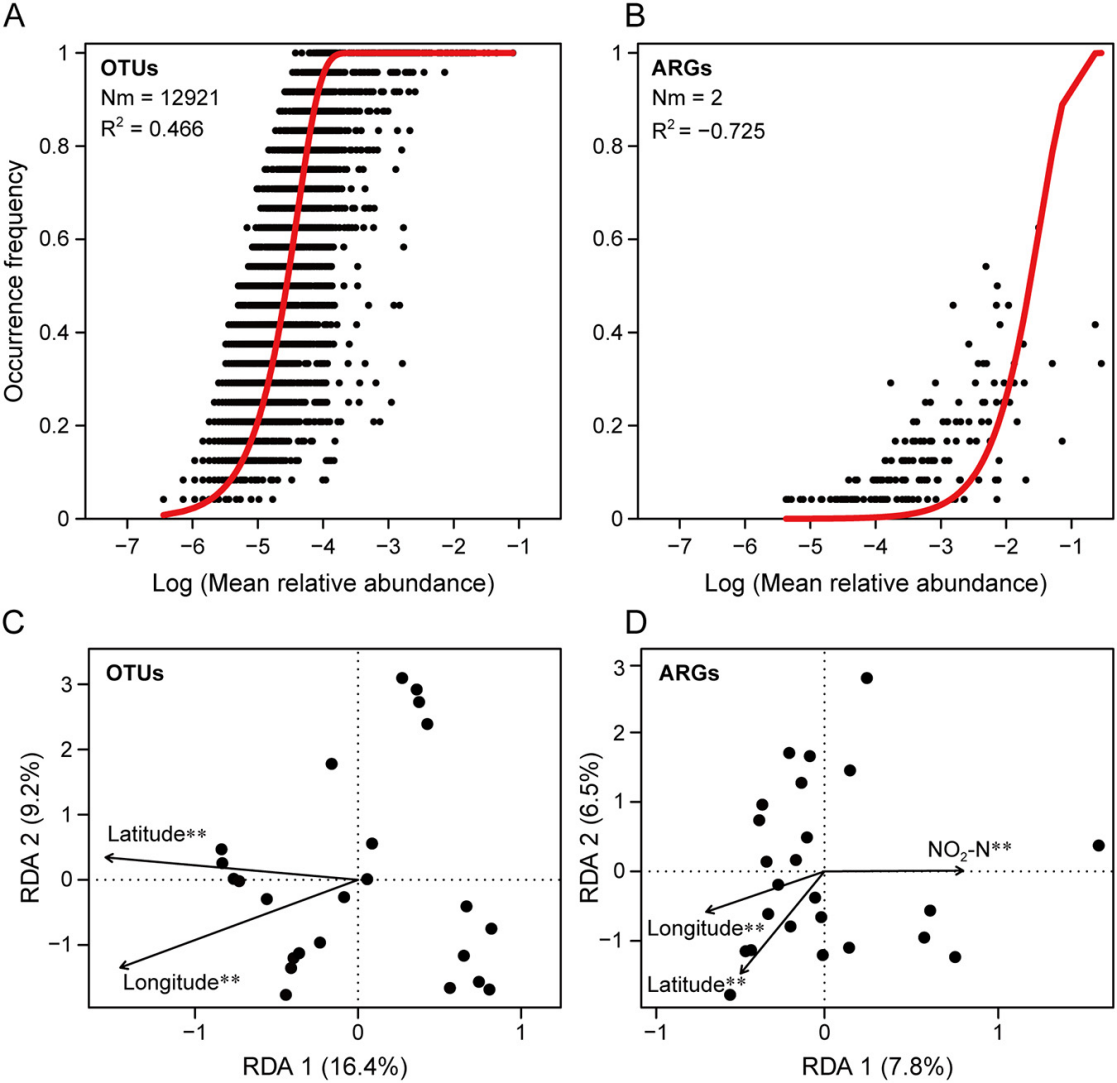
The assembly mechanisms of bacterial communities and ARG profiles. The fit of the neutral community model for (A) bacterial communities and (B) ARG profiles. Red lines represent the best fit for the neutral model. Nm indicates metacommunity size times immigration, and R^2^ indicates the fit to the neutral model. Note that the negative R^2^ value indicates no fit to the neutral model. Redundancy analysis (RDA) shows the relationship between environmental factors (including latitude and longitude) and bacterial communities (C), and the relationship between environmental factors and ARGs (D). NO_2_-N, nitrite nitrogen. Only factors with significant correlation are shown. **, *P* < 0.01.

We used the neutral community model (NCM) to evaluate the importance of stochastic processes underlying the distribution of bacterial communities and ARG profiles. The NCM explained 46.6% of spatial variation in bacterial community composition, but ARG profiles did not fit the NCM (the negative value of R^2^ meant no fit), indicating that stochastic or neutral processes did not play an important role in shaping the spatial distribution of ARG profiles (Fig. 3A and B). Redundancy analysis (RDA) further revealed that among 21 environmental variables analyzed, only longitude and latitude were significantly correlated with the composition of the bacterial community, while longitude, latitude, and nitrite nitrogen exhibited a significant correlation with the composition of ARG profiles (Fig. 3C and D). In addition, both longitude and latitude were negatively related to the richness of ARGs (Fig. S6). The α-diversity of the bacterial community was significantly correlated with water depth, nitrite nitrogen, total phosphorus, and oxidation-reduction potential (ORP), while the normalized abundance of ARGs was significantly correlated with ORP (Fig. S6). This indicates that the biogeographical patterns of bacterial communities and ARG profiles were driven by different ecological processes and mechanisms. The stochastic and deterministic processes simultaneously drove bacterial community assembly, while deterministic processes (e.g., effects of the local environmental conditions) mainly influenced the assembly of ARG profiles.

### Relationship between ARG profiles and bacterial communities.

**FIG 4:**
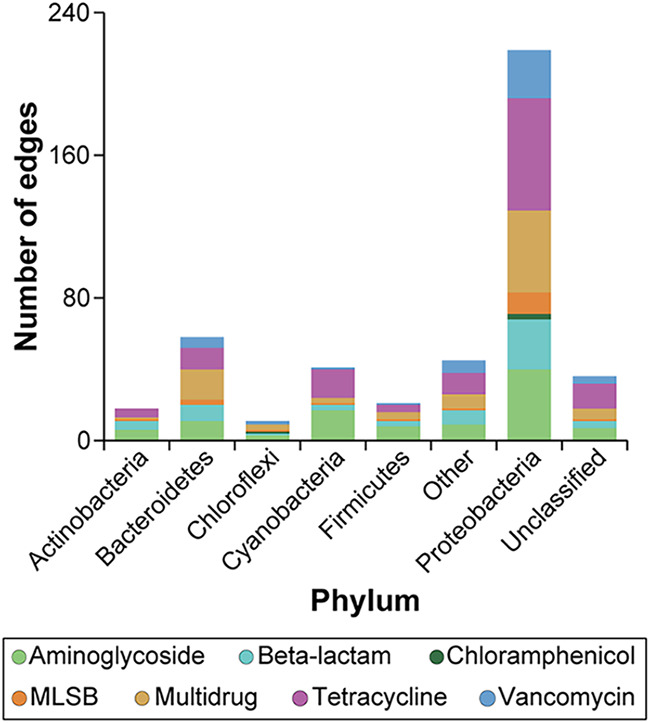
The number of edges (connections) in the co-occurrence network of bacterial OTUs and ARG subtypes. Only connections with a strong (Spearman’s correlation coefficient |r| ≥ 0.8) and significant (*P* < 0.01) correlation are presented in the network.

Co-occurrence patterns of bacterial taxa and ARG subtypes were established between 326 OTUs and 77 ARGs by network analysis and revealed that genes conferring resistance to aminoglycosides, beta-lactams, tetracyclines, and multidrug were significantly correlated with bacterial taxa (Fig. S7). The network consisted of 403 nodes and 449 edges (i.e., strong and significant correlations), the correlations were all positive and significant with coefficient values *r* > 0.8. The frequently connected nodes in the network were ARGs, especially genes encoding resistance to tetracyclines, vancomycin, multidrug, and aminoglycosides, which were defined as “hubs” of the module. Bacterial taxa belonging to the phylum Proteobacteria established linkages with most ARGs, followed by Bacteroidetes and Cyanobacteria (Fig. 4).

However, the similarities of bacterial community and ARG profile did not show a significant correlation (*r* = 0.084, *P* = 0.063), and the Procrustes test also indicated that bacterial community and ARG profile were not significantly correlated (*r* = 0.254, *P* = 0.442) (Fig. S8). Mantel test further revealed that only aminoglycoside, beta-lactam, chloramphenicol, MLSB, and tetracycline resistance genes were weakly and significantly related to Chloroflexi, Cyanobacteria, Proteobacteria, Actinobacteria, Cyanobacteria/Patescibacteria, respectively (Table S3).

### Co-occurrence pattern of ARGs and MGEs.

The absolute abundance of ARGs was positively correlated with the absolute abundance of MGEs (*r* = 0.523, *P* < 0.05), and the normalized abundance of ARGs was positively related to that of MGEs (*r* = 0.516, *P* < 0.05), elucidating an overall significant correlation between ARGs and MGEs (Fig. S6). The absolute abundance of the *intI1* gene was positively related to that of multidrug resistance genes, while the normalized abundance of the *intI1* gene did not show a significant relationship with any ARGs (Table S4 and S5). The co-occurrence patterns between ARGs and MGE marker genes indicated that both *IS613* and *tnpA-03* genes were significantly correlated with more resistance genes, while the *intI1* gene did not show any significant relationship with resistance genes (Table S6).

## DISCUSSION

### Distinct biogeographical patterns of ARG profiles and bacterial communities.

We investigated the biogeographical patterns of bacterial communities and ARG profiles in 24 inland water bodies in 5 provinces of China, including 19 drinking water supply reservoirs. The results indicate that the composition of bacterial communities and ARG profiles significantly correlated with latitude and longitude, and they showed a significant distance-decay relationship. Further, the richness of ARGs significantly decreased with increasing latitude (r = −0.615, *P* = 0.001), indicating the composition and richness of ARG profiles exhibited a biogeographical pattern. However, neither absolute nor normalized relative abundance of ARG profiles exhibited any biogeographical pattern. Similar to our results, previous studies in other environments reported that the abundance of ARGs did not show clear spatial patterns in wastewater treatment plants and estuaries (20, 27). Spatial distribution patterns of ARG abundance might rather be driven by the widespread use of diverse antibiotics, which exerted long-term selection or coselection pressure on ARGs in polluted environments. This indicates that ARG abundance is majorly driven by complex factors from local wastewaters (28, 29). In addition, according to previous studies which explored the abundance of ARGs using the same HT-qPCR approach, the richness (8–39) and abundance (2.22 × 10^6^ to 4.47 × 10^8^ copies/liter) of ARGs in the water bodies of this study was lower than that in river-reservoirs (81 to 213, 1.9 × 10^9^ to 2.6 × 10^11^ copies/liter), urban rivers (100 to 159, 3.61 × 10^10^ to 3.02 × 10^11^ copies/liter) and peri-urban river systems (46 to 154, 1.35 × 10^8^ to 1.16 × 10^9^ copies/liter) (30–32). This may be because the major water bodies of this study serve as sources of drinking water and were protected by local governments and thus exposed to much less impact through human activities compared to rivers in previous studies. The richness and abundance of ARGs in rural water bodies investigated by Liu et al. (3) were similar to the results of our study. Hence, this study provides a baseline and reference for the abundance and richness of ARGs in Chinese drinking water sources. Bacterial communities and ARG profiles exhibited distinct biogeographical patterns in the inland freshwaters of southeast China. The composition of bacterial communities showed high similarity among samples and those obtained from the same province clustered together, while the similarity of ARG profiles was relatively low and ARGs were significantly different among the sampled water bodies. These results suggest that bacteria may be dispersal-limited, while the distribution of antibiotic resistance genes may be shaped by more complex mechanisms. Similar to our results, Han et al. (33) reported the significantly higher distance-decay relationships of bacterial communities compared to those of ARGs in drinking water samples across a large geographical scale. These results indicate that spatial factors contributed more to bacterial community assembly, while ARG profiles were simultaneously shaped by local and regional environmental factors. Previous studies reported the differences in ARG profiles in global lake sediments and estuaries and suggested variations in observed ARGs were most likely related to the local clinical patterns of antibiotic prescriptions and human activities, including fecal pollution (27, 34, 35). Consistent with previous works (36, 37), the sulfonamide resistance gene *sul2* and aminoglycoside gene *aadA1* were frequently detected in our samples (Fig. S4). This is unsurprising as sulfonamides are the first antibiotic applied in the clinic, and aminoglycosides are also widely used in clinical settings since their discovery (38). The most frequently detected ARG class of multidrug resistance genes commonly referred to as genes in the current ARG database provide resistance to more than one class of antibiotics and encode general efflux pumps that provide low-level resistance to several antibiotics. They normally have additionally other general functions, such as efflux of metal cations, and they are most commonly not genes of high clinical relevance (13). Contrary to those ARG specific to a single antibiotic class usually causes high-level resistance up to clinically relevant antibiotic concentrations. Consequently, it is not surprising, that multidrug resistance efflux systems are the most found in low anthropogenic impact areas, while more specialized high-level resistance genes would be expected to dominate in highly anthropogenic impact environments areas (39–41).

Here, most of these high-level ARGs were detected only once in this study, and the dominant ARG classes exhibited significant differences between different water bodies. The distinct ARGs detected in inland waters at a large scale suggested that the monitoring of ARGs should be conducted according to local environmental conditions in different geographical regions. The observed great variation in ARG profiles, suggests that ARG profiles had unique patterns in different water bodies.

### Different assembly processes of ARG profiles and bacterial communities.

Bacterial communities and ARG profiles in inland waters followed different assembly mechanisms in this study. Stochastic and deterministic processes are two types of processes that affect the assembly of communities. The stochastic processes are neutral-based processes, including random changes in species birth, death, immigration, speciation, and limited dispersal, whereas the deterministic processes are niche-based processes, including the influence of abiotic and biotic factors on the community (42). The biogeographical patterns of bacterial communities were simultaneously driven by stochastic and deterministic processes, while the stochastic process did not contribute to the spatial distribution of ARG profiles (Fig. 3). The neutral theory claims that the assembly of microbial communities is governed by a stochastic process such as immigration, birth-death, and dispersal, while the niche theory highlights the importance of deterministic processes, including environmental variables and species interactions (42–44). A previous study has demonstrated the role of environmental factors, geographical distance, and neutral processes in shaping bacterial community composition in inland waters (45). In addition, previous studies in environments with less anthropogenic impact highlight that the temporal dynamics of ARG profiles fitted well to the neutral model, indicating they could be partly explained by stochastic processes (10, 46). However, our results suggested that the spatial distribution of ARG profiles did not fit the neutral model, most likely due to the different local environmental conditions and lack of human impact on the water bodies. Longitude, latitude, and nitrite nitrogen showed a significant correlation with ARG profiles (Fig. 3), with longitude and latitude being negatively correlated with the richness of ARGs (Fig. S6). Recent studies reported the contribution of spatial factors to the biogeographical patterns of ARGs (3), and nutrients have effects on the temporal variation of ARGs (46). The different assembly mechanisms of the biogeographical patterns of bacterial communities and ARG profiles can partly explain their distinct distribution patterns, but a large proportion of variation in ARG profiles remains not well explained. This might be due to some factors not being considered which relate to regional differences, including the concentration of antibiotic and heavy metal residues, local antibiotic usage, and other anthropogenic factors (e.g., human health and socio-economic factors) (4, 29). Therefore, the key drivers and mechanisms of the biogeographical patterns of ARG profiles warrant further study.

### Weak correlation between ARG profiles and bacterial communities.

Our data indicate that the bacterial community composition in the 24 freshwater bodies had only little effect on the distribution of ARG profiles. Recently, Ju et al. (11) found that the antibiotic resistomes in wastewaters were strongly shaped by bacterial communities, and Luo et al. (47) reported similar distribution patterns of bacterial communities and ARG profiles in full-scale biogas reactors. Zhou et al. (32) investigated the distribution of bacterial communities and ARG profiles in urban river systems which contained diverse pollutants at high concentrations. They also suggested that bacterial communities significantly correlated with ARG profiles and explained most of the variation in ARG composition. Consistent with our study, previous studies documented the inconsistent patterns and weak correlations between bacterial community composition and ARG profiles in drinking water and Chinese estuaries (27, 48). These results may be attributed to the low levels of pollution with selective agents and low-intensity human activities in the reservoir watersheds of this study. In general, antibiotic pollution directly selects for ARGs at low, environmentally relevant concentrations (49, 50). Additionally, other pollutants such as nonantibiotic pharmaceutical residues, heavy metal ions, or biocides have been shown to coselect for ARGs or to promote horizontal gene transfer of ARGs encoded on MGEs (51–54). Because most reservoirs in this study are sources of drinking water and are protected by local government regulations, the absence of these pollutants results in low selection pressure on the resistomes, and bacterial species without ARGs can survive in the diverse niches within these environments with high fitness.

MGEs can transfer ARGs between different bacterial species, usually coexist with ARGs, and play a key role in their dissemination (30, 31). In this study, the absolute abundance of MGEs and ARGs showed a significant and positive correlation (Fig. S6 and Table S4), indicating that, even in environments with relatively low human impact, MGEs might play a key role in the distribution of ARGs (3). Further, the absolute abundance of *intI1* showed a significant and positive correlation with the absolute abundance of multidrug resistance genes. However, compared to other studies, *intI1* only played a minor role as an explanatory variable for the abundance of other classes of resistance genes and was not even detected in all samples. This coincides with the fact that drinking water reservoirs experience low-intensity human impact because *intI1* is regularly proposed to be an indicator of anthropogenic pollution (55). The low levels of *intI1* confirm the low impact of anthropogenic pollution on the drinking water sources in this study. However, other MGEs, less connected to anthropogenic pollution, were proven to still play an important role in explaining the spatial distribution patterns of ARGs (56).

This study uncovered the distinct biogeographical patterns of bacterial communities and ARG profiles in inland waters of southeast China under low-human impact at a large scale. The composition of ARG profiles showed a greater difference among samples than bacterial community composition, while the bacterial and ARG absolute abundances did not exhibit any strongly spatial pattern along latitude or longitude. The ARGs were significantly different among samples, indicating the impact of local factors on ARGs or complex dynamics of ARGs. We further found the biogeographical patterns of bacterial communities were simultaneously driven by stochastic and deterministic processes, whereas the geographical pattern of ARG profiles could not be explained by stochastic processes. A weak correlation between bacterial community composition and ARG profiles was identified. This study improved our understanding of ARG distribution in inland waters with emphasis on drinking water supply reservoirs at a large scale, therefore providing baseline information needed for future monitoring and risk assessment of ARGs in drinking water sources.

## MATERIALS AND METHODS

### Study sites and sampling.

Water samples were collected from the epilimnion (surface waters) in 24 reservoirs across 5 provinces of southeast China (Fig. S1 and Table S1) during July and August in 2018. The sampling procedures can be found in a previous study (3). Normally, sampling sites were located at the center of each reservoir to ensure that samples were as representative and as comparable as possible. At these locations, the maximum distance from the inflow and outflow of the reservoirs is gained. The latitude of these reservoirs ranged from 18 to 30° N, across approximately 1800 km. Water samples were taken using a sterile 5-liter polypropylene bottle. All samples were kept in the dark and filtered within 2 h of sampling. Water samples were prefiltered through a 200 μm mesh and then filtered through a 0.22 μm polycarbonate filter (47 mm diameter, Millipore, Billerica, MA, USA) using a vacuum filtration system. To collect sufficient bacterial biomass, we normalized the filtration time of each sample to about 40 min. The total filtrated water volume ranged from 200 mL for hypereutrophic waters to 750 mL for oligotrophic waters. Filters were then placed in sterilized tubes and stored at −80°C until DNA extraction.

Water depth and transparency of sampling sites were measured as described in our previous study (23). Water temperature, pH, dissolved oxygen, chlorophyll-*a*, turbidity, electrical conductivity, salinity, and ORP were measured *in situ* with a Hydrolab DS5 multiparameter water quality analyzer (Hach Company, Loveland, CO, USA). Suspended solids, total carbon, total organic carbon, total nitrogen, ammonium nitrogen, nitrate nitrogen, nitrite nitrogen, total phosphorus, and phosphate phosphorus were measured according to standard methods (57).

### DNA extraction and quantitative PCR.

Total DNA from the water samples was extracted from filters using the FastDNA SPIN kit (MP Biomedicals, Santa Ana, CA, USA) according to the manufacturer’s protocol. DNA quality and concentration were assessed using a NanoDrop 1000 spectrophotometer (Thermo Scientific, Waltham, MA, USA).

In total, 296 genes were analyzed by HT-qPCR using the Wafergen SmartChip real-time quantitative PCR platform (Wafergen Biosystems, Fremont, CA, USA). These included the 16S rRNA gene, 285 resistance genes, 8 transposase genes, the class I integron-integrase gene (*intI1*), and the clinical class 1 integron-integrase gene (*cIntI*) (10). Detailed information regarding the primer sets and PCR protocols were described in our previous study (Table S1 in Guo et al. [10]). For each sample, three technical replicates were performed and a nontemplate negative-control was also included for each of the HT-qPCR assays. For quality control any wells with multiple melting peaks and/or amplification efficiency <1.8 or >2.2 were discarded. Further, the HT-qPCR threshold cycle (Ct) of 31 was used as the detection limit, and only samples with three replicates that simultaneously reached a Ct <31 were regarded as positive.

The absolute copy number of the 16S rRNA gene was quantified by real-time quantitative PCR (qPCR) using a Lightcycler 480 instrument (Roche, Basel, Switzerland). To calculate the absolute abundance of the 16S rRNA gene, we used a six-point calibration curve from a 10-fold dilution of a standard plasmid containing a cloned and sequenced 16S rRNA fragment with the highest concentration starting at 3 × 10^10^ copies/liter. PCRs were performed in triplicates with three negative controls following our previous study (46). Only standard curves with an amplification efficiency of 90% to 110% and R^2^ ≥ 0.99 and single melting peaks were accepted. Similarly, the amplification efficiency of all samples was assessed to rule out any potential PCR inhibition and for all samples, the amplification efficiency was between 90% and 110%.

The relative copy number of the 16S rRNA gene from HT-qPCR was significantly correlated with the absolute copy number from qPCR. Therefore, the relative copy number of ARGs and MGEs generated from HT-qPCR was transformed to absolute abundance according to equation (58): $\text{AR}{\text{G}}_{\text{HT}-\text{qPCR }}\left(\text{or }{\mathrm{MGE}}_{\text{HT}-\text{qPCR}}\right)/\text{16}{\text{S}}_{\text{HT}-\text{qPCR}}=\text{AR}{\text{G}}_{\text{absolute }}\left(\text{or }{\mathrm{MGE}}_{\text{absolute}}\right)/\text{16}{\text{S}}_{\text{qPCR}}.$

### High-throughput sequencing.

Sequencing of the 16S rRNA gene, to characterize the bacterial communities, was performed at Novogene Bioinformatics Technology (Beijing, China) on the Illumina HiSeq2500 (PE250) platform using the PCR primer set (341F-forward: CCTAYGGGRBGCASCAG, 806R-reverse: GGACTACNVGGGTWTCTAAT) (59, 60) targeting the V3–V4 region (61). Amplicons were barcoded (at 5′ ends of the primers) during the amplification step. Thereafter, PCR products were purified. Then sequencing was performed according to the Novogene Bioinformatics Technology standardized protocol, which includes a no template control to account for potential reagent contamination in each run. No reagent contamination was detected in our samples.

After sequenced raw reads were merged in MOTHUR v1.39.0 (62) using the make.contigs() command in MOTHUR with pdiffs = 5, bdiffs = 1 was used to obtain merged fasta and quality files. Sequence quality control and OTU calling were performed in the USEARCH environment (63). First, dereplication was performed using the vsearch command (minuniquesize = 8) to keep exclusively high-quality sequences, thereafter the unoise3 algorithm was used with the default setting (minsize = 8) to obtain OTUs from those sequences at a 97% similarity level (64). OTU representative sequences were then classified using USEARCH (sintax) against the SILVA 132 database (65). All eukaryotic, chloroplast, archaeal, mitochondrial, chimeras, and unknown sequences were excluded. After this step the number of cleaned and high-quality sequences across all samples ranged from 115,984 to 286,812, hence we normalized the sequencing depth to 115,984 sequences for each sample. These sequences were clustered into 4971 OTUs.

### Statistics.

The data of bacterial community composition, ARG profiles, and environmental variables (except pH) were log-transformed before analyses to improve normality. The Shannon-Wiener index and Bray-Curtis similarity were calculated at the OTU level using R v3.6.1 with the vegan package (66). PCoA and analysis of similarity (ANOSIM) were carried out to explore compositional differences in Bray-Curtis similarity between samples. To compare the number of detected genes, we constructed a Venn diagram using the “Venn-Diagram” package in the R environment (67).

In addition, we calculated the pairwise Spearman’s correlations at the OTU level with the picante package to reveal the relationship between bacterial taxa and ARGs (68). The correlation coefficients |*ρ*| ≥ 0.8 between bacterial taxa and ARGs, |*ρ*| ≥ 0.6 between MGEs and ARGs, and *P* < 0.01 were considered statistically significant, and network information was constructed using the Gephi v8.2. The Procrustes test was performed at the OTU level within the vegan package to test the correlation between bacterial communities and ARG profiles. Mantel tests were calculated at the OTU level using R v3.6.1 with the vegan package to reveal Spearman’s correlations between bacterial OTUs and ARG classes.

The neutral community model was calculated at the OTU level to test the importance of stochastic processes in shaping the assembly of bacterial communities and resistomes (21, 44), with the Hmisc, stats4, and minpack.lm packages in R v3.6.1 (67). We performed RDA at the OTU level with the vegan package to explore the relationship between environmental factors and the compositions of bacterial communities and ARG profiles, respectively. We used forward selection to choose significant environmental factors (*P* < 0.05) before the RDA.

### Data availability.

The raw data for the 16S rRNA gene has been deposited in the National Omics Data Encyclopedia database under the Project ID: OEP001334. These raw sequences were also stored in the NCBI sequence read archive (SRA) database under the BioProject number PRJNA694227.

## References

[1] D'Costa VM, King CE, Kalan L, Morar M, Sung WWL, Schwarz C, Froese D, Zazula G, Calmels F, Debruyne R, Golding GB, Poinar HN, Wright GD. 2011. Antibiotic resistance is ancient. Nature 477:457–461. doi:10.1038/nature10388. 21881561

[2] Laxminarayan R. 2014. Antibiotic effectiveness: balancing conservation against innovation. Science 345:1299–1301. doi:10.1126/science.1254163. 25214620

[3] Liu LM, Su JQ, Guo YY, Wilkinson DM, Liu ZW, Zhu YG, Yang J. 2018. Large-scale biogeographical patterns of bacterial antibiotic resistome in the waterbodies of China. Environ Int 117:292–299. doi:10.1016/j.envint.2018.05.023. 29891393

[4] Hendriksen SR, Munk P, Njage P, van Bunnik B, McNally L, Lukjancenko O, Röder T, Nieuwenhuijse D, Pedersen SK, Kjeldgaard J, Kaas RS, Clausen PTLC, Vogt JK, Leekitcharoenphon P, van de Schans MGM, Zuidema T, de Roda Husman AM, Rasmussen S, Petersen B, Amid C, Cochrane G, Sicheritz-Ponten T, Schmitt H, Alvarez JRM, Aidara-Kane A, Pamp SJ, Lund O, Hald T, Woolhouse M, Koopmans MP, Vigre H, Petersen TN, Aarestrup FM, Global Sewage Surveillance project consortium. 2019. Global monitoring of antimicrobial resistance based on metagenomics analyses of urban sewage. Nat Commun 10:1124. doi:10.1038/s41467-019-08853-3. 30850636 PMC6408512

[5] Martínez JL. 2008. Antibiotics and antibiotic resistance genes in natural environments. Science 321:365–367. doi:10.1126/science.1159483. 18635792

[6] Pruden A, Pei R, Storteboom H, Carlson KH. 2006. Antibiotic resistance genes as emerging contaminants: studies in northern Colorado. Environ Sci Technol 40:7445–7450. doi:10.1021/es060413l. 17181002

[7] Chen J, McIlroy SE, Archana A, Baker DM, Panagiotou G. 2019. A pollution gradient contributes to the taxonomic, functional, and resistome diversity of microbial communities in marine sediments. Microbiome 7:104. doi:10.1186/s40168-019-0714-6. 31307536 PMC6632204

[8] Gao M, Qiu T, Sun Y, Wang X. 2018. The abundance and diversity of antibiotic resistance genes in the atmospheric environment of composting plants. Environ Int 116:229–238. doi:10.1016/j.envint.2018.04.028. 29698899

[9] van Goethem MW, Pierneef R, Bezuidt OKI, Van De Peer Y, Cowan DA, Makhalanyane TP. 2018. A reservoir of ‘historical’ antibiotic resistance genes in remote pristine Antarctic soils. Microbiome 6:40. doi:10.1186/s40168-018-0424-5. 29471872 PMC5824556

[10] Guo YY, Liu M, Liu LM, Liu X, Chen HH, Yang J. 2018. The antibiotic resistome of free-living and particle-attached bacteria under a reservoir cyanobacterial bloom. Environ Int 117:107–115. doi:10.1016/j.envint.2018.04.045. 29734061

[11] Ju F, Beck K, Yin X, Maccagnan A, McArdell CS, Singer HP, Johnson DR, Zhang T, Bürgmann H. 2019. Wastewater treatment plant resistomes are shaped by bacterial composition, genetic exchange, and upregulated expression in the effluent microbiomes. ISME J 13:346–360. doi:10.1038/s41396-018-0277-8. 30250051 PMC6331547

[12] Amos GCA, Zhang L, Hawkey PM, Gaze WH, Wellington EM. 2014. Functional metagenomic analysis reveals rivers are a reservoir for diverse antibiotic resistance genes. Vet Microbiol 171:441–447. doi:10.1016/j.vetmic.2014.02.017. 24636906

[13] Marti E, Variatza E, Balcazar JL. 2014. The role of aquatic ecosystems as reservoirs of antibiotic resistance. Trends Microbiol 22:36–41. doi:10.1016/j.tim.2013.11.001. 24289955

[14] Rodriguez-Mozaz S, Chamorro S, Marti E, Huerta B, Gros M, Sànchez-Melsió A, Borrego CM, Barceló D, Balcázar JL. 2015. Occurrence of antibiotics and antibiotic resistance genes in hospital and urban wastewaters and their impact on the receiving river. Water Res 69:234–242. doi:10.1016/j.watres.2014.11.021. 25482914

[15] Brauman KA, Daily GC, Duarte TK, Mooney HA. 2007. The nature and value of ecosystem services: an overview highlighting hydrologic services. Annu Rev Environ Resour 32:67–98. doi:10.1146/annurev.energy.32.031306.102758.

[16] Castello L, Macedo MN. 2016. Large-scale degradation of Amazonian freshwater ecosystems. Glob Chang Biol 22:990–1007. doi:10.1111/gcb.13173. 26700407

[17] Yang JR, Lv H, Isabwe A, Liu LM, Yu XQ, Chen HH, Yang J. 2017. Disturbance-induced phytoplankton regime shifts and recovery of cyanobacteria dominance in two subtropical reservoirs. Water Res 120:52–63. doi:10.1016/j.watres.2017.04.062. 28478295

[18] Baquero F, Martínez JL, Cantón R. 2008. Antibiotics and antibiotic resistance in water environments. Curr Opin Biotechnol 19:260–265. doi:10.1016/j.copbio.2008.05.006. 18534838

[19] Czekalski N, Radhika S, Birtel J, Matthews B, Bürgmann H. 2015. Does human activity impact the natural antibiotic resistance background? Abundance of antibiotic resistance genes in 21 Swiss lakes. Environ Int 81:45–55. doi:10.1016/j.envint.2015.04.005. 25913323

[20] Cacace D, Fatta-Kassinos D, Manaia CM, Cytryn E, Kreuzinger N, Rizzo L, Karaolia P, Schwartz T, Alexander J, Merlin C, Garelick H, Schmitt H, de Vries D, Schwermer CU, Meric S, Ozkal CB, Pons M-N, Kneis D, Berendonk TU. 2019. Antibiotic resistance genes in treated wastewater and in the receiving water bodies: a pan-European survey of urban settings. Water Res 162:320–330. doi:10.1016/j.watres.2019.06.039. 31288142

[21] Peng F, Guo YY, Isabwe A, Chen HH, Wang YM, Zhang YP, Zhu ZX, Yang J. 2020. Urbanization drives riverine bacterial antibiotic resistome more than taxonomic community at watershed scale. Environ Int 137:105524. doi:10.1016/j.envint.2020.105524. 32036121

[22] Hernando-Amado S, Coque TM, Baquero F, Martinez JL. 2019. Defining and combating antibiotic resistance from One Health and global health perspectives. Nat Microbiol 4:1432–1442. doi:10.1038/s41564-019-0503-9. 31439928

[23] Liu LM, Yang J, Yu Z, Wilkinson DM. 2015. The biogeography of abundant and rare bacterioplankton in the lakes and reservoirs of China. ISME J 9:2068–2077. doi:10.1038/ismej.2015.29. 25748371 PMC4542038

[24] Haggerty JM, Dinsdale EA, Davies J. 2017. Distinct biogeographical patterns of marine bacterial taxonomy and functional genes. Global Ecol Biogeogr 26:177–190. doi:10.1111/geb.12528.

[25] Angermeyer A, Crosby SC, Huber JA. 2016. Decoupled distance-decay patterns between dsrA and 16S rRNA genes among salt marsh sulfate-reducing bacteria. Environ Microbiol 18:75–86. doi:10.1111/1462-2920.12821. 25727503

[26] Ruiz-González C, Niño-García JP, Lapierre JF, del Giorgio PA. 2015. The quality of organic matter shapes the functional biogeography of bacterioplankton across boreal freshwater ecosystems. Glob Ecol Biogeogr 24:1487–1498. doi:10.1111/geb.12356.

[27] Zhu YG, Zhao Y, Li B, Huang CL, Zhang SY, Yu S, Chen YS, Zhang T, Gillings MR, Su JQ. 2017. Continental-scale pollution of estuaries with antibiotic resistance genes. Nat Microbiol 2:16270. doi:10.1038/nmicrobiol.2016.270. 28134918

[28] Bengtsson-Palme J, Kristiansson E, Larsson DGJ. 2018. Environmental factors influencing the development and spread of antibiotic resistance. FEMS Microbiol Rev 42:68–80. doi:10.1093/femsre/fux053. PMC5812547 29069382

[29] Lopatkin AJ, Huang S, Smith RP, Srimani JK, Sysoeva TA, Bewick S, Karig D, You L. 2016. Antibiotics as a selective driver for conjugation dynamics. Nat Microbiol 1:16044. doi:10.1038/nmicrobiol.2016.44. 27572835 PMC5010019

[30] Chen Y, Su JQ, Zhang J, Li P, Chen H, Zhang B, Gin KYH, He Y. 2019. High-throughput profiling of antibiotic resistance gene dynamic in a drinking water river-reservoir system. Water Res 149:179–189. doi:10.1016/j.watres.2018.11.007. 30447523

[31] Zheng J, Zhou Z, Wei Y, Chen T, Feng W, Chen H. 2018. High-throughput profiling of seasonal variations of antibiotic resistance gene transport in a peri-urban river. Environ Int 114:87–94. doi:10.1016/j.envint.2018.02.039. 29499451

[32] Zhou ZC, Zheng J, Wei YY, Chen T, Dahlgren RA, Shang X, Chen H. 2017. Antibiotic resistance genes in an urban river as impacted by bacterial community and physicochemical parameters. Environ Sci Pollut Res 24:23753–23762. doi:10.1007/s11356-017-0032-0. 28864929

[33] Han Z, Zhang Y, An W, Lu J, Hu J, Yang M. 2020. Antibiotic resistomes in drinking water sources across a large geographical scale: multiple drivers and co-occurrence with opportunistic bacterial pathogens. Water Res 183:116088. doi:10.1016/j.watres.2020.116088. 32622239

[34] Chen H, Jing L, Yao Z, Meng F, Teng Y. 2019. Prevalence, source and risk of antibiotic resistance genes in the sediments of Lake Tai (China) deciphered by metagenomic assembly: a comparison with other global lakes. Environ Int 127:267–275. doi:10.1016/j.envint.2019.03.048. 30928850

[35] Karkman A, Parnanen K, Larsson DG. 2019. Fecal pollution can explain antibiotic resistance gene abundances in anthropogenically impacted environments. Nat Commun 10:80. doi:10.1038/s41467-018-07992-3. 30622259 PMC6325112

[36] Che Y, Xia Y, Liu L, Li A, Yang Y, Zhang T. 2019. Mobile antibiotic resistome in wastewater treatment plants revealed by Nanopore metagenomic sequencing. Microbiome 7:44. doi:10.1186/s40168-019-0663-0. 30898140 PMC6429696

[37] Yang Y, Song W, Lin H, Wang W, Du L, Xing W. 2018. Antibiotics and antibiotic resistance genes in global lakes: a review and meta-analysis. Environ Int 116:60–73. doi:10.1016/j.envint.2018.04.011. 29653401

[38] Forge A, Schacht J. 2000. Aminoglycoside antibiotics. Audiol Neurootol 5:3–22. doi:10.1159/000013861. 10686428

[39] Mazhar SH, Li X, Rashid A, Su JM, Xu J, Brejnrod AD, Su JQ, Wu Y, Zhu YG, Zhou SG, Feng R, Rensing C. 2021. Co-selection of antibiotic resistance genes, and mobile genetic elements in the presence of heavy metals in poultry farm environments. Sci Total Environ 755:142702. doi:10.1016/j.scitotenv.2020.142702. 33049532

[40] Sevillano M, Dai Z, Calus S, Bautista-de Los Santos QM, Eren AM, van der Wielen PW, Ijaz UZ, Pinto AJ. 2020. Differential prevalence and host-association of antimicrobial resistance traits in disinfected and non-disinfected drinking water systems. Sci Total Environ 749:141451. doi:10.1016/j.scitotenv.2020.141451. 32836121

[41] Sun Y, Cao N, Duan C, Wang Q, Ding C, Wang J. 2021. Selection of antibiotic resistance genes on biodegradable and non-biodegradable microplastics. J Hazard Mater 409:124979. doi:10.1016/j.jhazmat.2020.124979. 33421879

[42] Hubbell SP. 2001. The unified neutral theory of biodiversity and biogeography. Princeton University Press, Princeton. 10.1016/j.tree.2011.03.024 21561679

[43] Dumbrell AJ, Nelson M, Helgason T, Dytham C, Fitter AH. 2010. Relative roles of niche and neutral processes in structuring a soil microbial community. ISME J 4:337–345. doi:10.1038/ismej.2009.122. 19924158

[44] Sloan WT, Lunn M, Woodcock S, Head LM, Nee S, Curtis TP. 2006. Quantifying the roles of immigration and chance in shaping prokaryote community structure. Environ Microbiol 8:732–740. doi:10.1111/j.1462-2920.2005.00956.x. 16584484

[45] Lindström ES, Langenheder S. 2012. Local and regional factors influencing bacterial community assembly. Environ Microbiol Rep 4:1–9. doi:10.1111/j.1758-2229.2011.00257.x. 23757223

[46] Fang P, Peng F, Gao X, Xiao P, Yang J. 2019. Decoupling the dynamics of bacterial taxonomy and antibiotic resistance function in a subtropical urban reservoir as revealed by high-frequency sampling. Front Microbiol 10:1448. doi:10.3389/fmicb.2019.01448. 31312186 PMC6614491

[47] Luo G, Li B, Li LG, Zhang T, Angelidaki I. 2017. Antibiotic resistance genes and correlations with microbial community and metal resistance genes in full-scale biogas reactors as revealed by metagenomic analysis. Environ Sci Technol 51:4069–4080. doi:10.1021/acs.est.6b05100. 28272884

[48] Ma L, Li B, Jiang XT, Wang YL, Xia Y, Li AD, Zhang T. 2017. Catalogue of antibiotic resistome and host-tracking in drinking water deciphered by a large scale survey. Microbiome 5:154. doi:10.1186/s40168-017-0369-0. 29179769 PMC5704573

[49] Gullberg E, Cao S, Berg OG, Ilbäck C, Sandegren L, Hughes D, Andersson DI. 2011. Selection of resistant bacteria at very low antibiotic concentrations. PLoS Pathog 7:e1002158. doi:10.1371/journal.ppat.1002158. 21811410 PMC3141051

[50] Klümper U, Recker M, Zhang L, Yin X, Zhang T, Buckling A, Gaze WH. 2019. Selection for antimicrobial resistance is reduced when embedded in a natural microbial community. ISME J 13:2927–2937. doi:10.1038/s41396-019-0483-z. 31384011 PMC6864104

[51] Jutkina J, Marathe NP, Flach CF, Larsson DGJ. 2018. Antibiotics and common antibacterial biocides stimulate horizontal transfer of resistance at low concentrations. Sci Total Environ 616–617:172–178. doi:10.1016/j.scitotenv.2017.10.312. 29112840

[52] Klümper U, Dechesne A, Riber L, Brandt KK, Gulay A, Sørensen SJ, Smets BF. 2017. Metal stressors consistently modulate bacterial conjugal plasmid uptake potential in a phylogenetically conserved manner. ISME J 11:152–165. doi:10.1038/ismej.2016.98. 27482924 PMC5097465

[53] Pal C, Bengtsson-Palme J, Kristiansson E, Larsson DGJ. 2015. Co-occurrence of resistance genes to antibiotics, biocides and metals reveals novel insights into their co-selection potential. BMC Genomics 16:1–14. doi:10.1186/s12864-015-2153-5. 26576951 PMC4650350

[54] Wang Y, Lu J, Mao L, Li J, Yuan Z, Bond PL, Guo J. 2019. Antiepileptic drug carbamazepine promotes horizontal transfer of plasmid-borne multi-antibiotic resistance genes within and across bacterial genera. ISME J 13:509–522. doi:10.1038/s41396-018-0275-x. 30291330 PMC6331567

[55] Gillings MR, Gaze WH, Pruden A, Smalla K, Tiedje JM, Zhu Y-G. 2015. Using the class 1 integron-integrase gene as a proxy for anthropogenic pollution. ISME J 9:1269–1279. doi:10.1038/ismej.2014.226. 25500508 PMC4438328

[56] Lekunberri I, Balcázar JL, Borrego CM. 2018. Metagenomic exploration reveals a marked change in the river resistome and mobilome after treated wastewater discharges. Environ Pollut 234:538–542. doi:10.1016/j.envpol.2017.12.001. 29220785

[57] Liu LM, Yang J, Yu XQ, Chen GJ, Yu Z. 2013. Patterns in the composition of microbial communities from a subtropical river: effects of environmental, spatial and temporal factors. PLoS One 8:e81232. doi:10.1371/journal.pone.0081232. 24244735 PMC3828266

[58] Ouyang WY, Huang FY, Zhao Y, Li H, Su JQ. 2015. Increased levels of antibiotic resistance in urban stream of Jiulongjiang River, China. Appl Microbiol Biotechnol 99:5697–5707. doi:10.1007/s00253-015-6416-5. 25661810

[59] Apprill A, Mcnally S, Parsons R, Weber L. 2015. Minor revision to V4 region SSU rRNA 806R gene primer greatly increases detection of SAR11 bacterioplankton. Aquat Microb Ecol 75:129–137. doi:10.3354/ame01753.

[60] Yu Y, Lee C, Kim J, Hwang S. 2005. Group-specific primer and probe sets to detect methanogenic communities using quantitative real-time polymerase chain reaction. Biotechnol Bioeng 89:670–679. doi:10.1002/bit.20347. 15696537

[61] Nyirabuhoro P, Gao XF, Ndayishimiye JC, Xiao P, Mo YY, Ganjidoust H, Yang J. 2021. Responses of abundant and rare bacterioplankton to temporal change in a subtropical urban reservoir. FEMS Microbiol Ecol 97:fiab036. doi:10.1093/femsec/fiab036. 33755730

[62] Schloss PD, Westcott SL, Ryabin T, Hall JR, Hartmann M, Hollister EB, Lesniewski RA, Oakley BB, Parks DH, Robinson CJ, Sahl JW, Stres B, Thallinger GG, Van Horn DJ, Weber CF. 2009. Introducing mothur: open-source, platform-independent, community-supported software for describing and comparing microbial communities. Appl Environ Microbiol 75:7537–7541. doi:10.1128/AEM.01541-09. 19801464 PMC2786419

[63] Edgar R. 2010. Usearch. Lawrence Berkeley National Lab. (LBNL), Berkeley, CA, USA.

[64] Edgar RC. 2010. Search and clustering orders of magnitude faster than BLAST. Bioinformatics 26:2460–2461. doi:10.1093/bioinformatics/btq461. 20709691

[65] DeSantis TZ, Hugenholtz P, Larsen N, Rojas M, Brodie EL, Keller K, Huber T, Dalevi D, Hu P, Andersen GL. 2006. Greengenes, a chimera-checked 16S rRNA gene database and workbench compatible with ARB. Appl Environ Microbiol 72:5069–5072. doi:10.1128/AEM.03006-05. 16820507 PMC1489311

[66] Oksanen J, Blanchet FG, Friendly M, Kindt R, Legendre P, McGlinn D, Minchin PR, O’Hara RB, Simpson GL, Solymos P, Stevens MHM, Szoecs E, Wagner H. 2020. vegan: Community ecology package. R package version 2.5-7. https://CRAN.R-project.org/package=vegan.

[67] R Core Team. 2020. R: A Language and Environment for Statistical Computing. R Foundation for Statistical Computing, Vienna, Austria. https://www.r-project.org.

[68] Kembel SW, Cowan PD, Helmus MR, Cornwell WK, Morlon H, Ackerly DD, Blomberg SP, Webb CO. 2010. Picante: R tools for integrating phylogenies and ecology. Bioinformatics 26:1463–1464. doi:10.1093/bioinformatics/btq166. 20395285

