# Supplementary material for: Biogeographical Patterns of Bacterial Communities and Their Antibiotic Resistomes in the Inland Waters of Southeast China
Source: Microbiol Spectr. 2022 Jun 23;10(4):e00406-22. doi: 10.1128/spectrum.00406-22 (PMC9430403; doi:10.1128/spectrum.00406-22)
Supplement: Supplemental file 1 — Supplemental material. Download spectrum.00406-22-s0001.pdf, PDF file, 1.4 MB [file spectrum.00406-22-s0001.pdf]

Supplementary information of the article:

Biogeographical patterns of bacterial communities and their antibiotic resistomes in the inland waters of southeast China

Peiju Fang[a,b,c,d], Peng Xiao[a], Fengjiao Tan[a,b], Yuanyuan Mo[a,b], Huihuang Chen[a,b,d], Uli Klümper[c], Thomas U. Berendonk[c], Jun Yang[a,d,*]

[a] *Aquatic Ecohealth Group, Fujian Key Laboratory of Watershed Ecology, Ningbo Observation and Research Station, Key Laboratory of Urban Environment and Health, Institute of Urban Environment, Chinese Academy of Sciences, Xiamen 361021, China*

[b] *University of Chinese Academy of Sciences, Beijing 100049, China*

[c] *Institute of Hydrobiology, Technical University of Dresden, Dresden 01217, Germany*

[d] *Zhejiang Key Laboratory of Urban Environmental Processes and Pollution Control, CAS Haixi Industrial Technology Innovation Center in Beilun, Ningbo 315830, China*

***Corresponding author:**

E-mail: jyang@iue.ac.cn (Jun Yang)

This supplementary information contains:

- 15 Pages
- 8 Figures
- 6 Tables

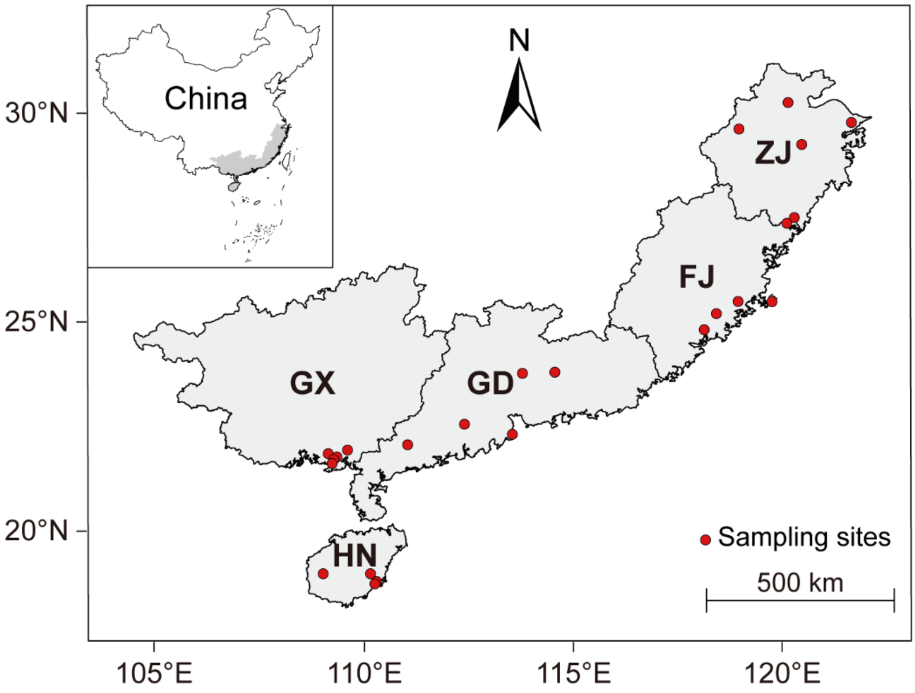

Fig. S1. Location of 24 sampling reservoirs in five provinces of southeast China. ZJ: Zhejiang province; FJ: Fujian province; GD: Guangdong province; GX: Guangxi province; HN: Hainan province.

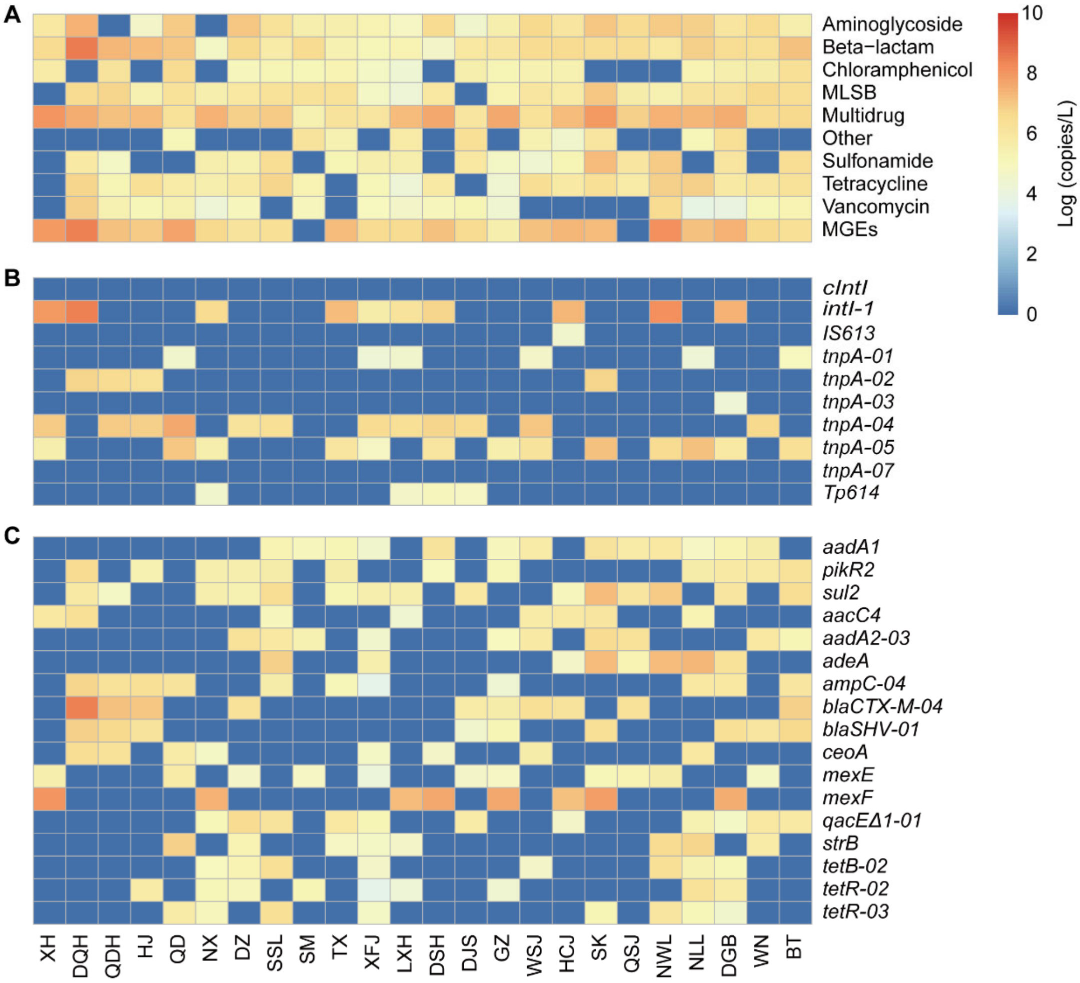

Fig. S2. Absolute abundance of antibiotic resistance genes (ARGs) and mobile genetic elements (MGEs) in 24 reservoirs. Profiling of **(A)** ARGs and MGEs, (**B**) detected MGEs and (**C**) core ARGs based on the absolute abundance [log(copies/L)], ranging from low (blue) to high (red) values. MLSB, macrolide-lincosamide-streptogramin B. For detailed abbreviation information of each reservoir see Table S1.

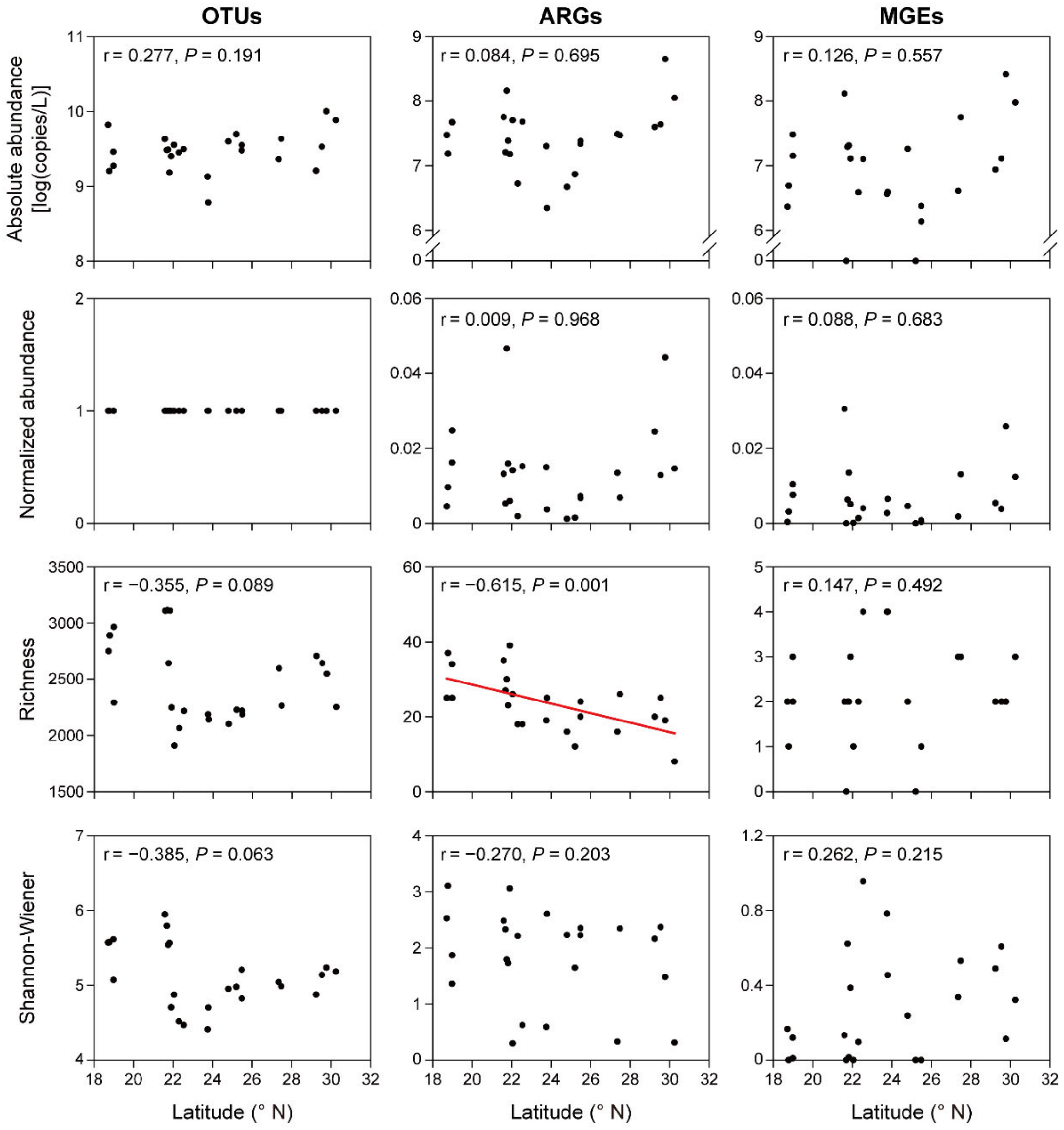

Fig. S3. The abundance, richness and diversity of bacterial communities, antibiotic resistance genes (ARGs) and mobile genetic elements (MGEs) profiles change along latitude gradient. Only significant Spearman's correlation is showed with fitted line.

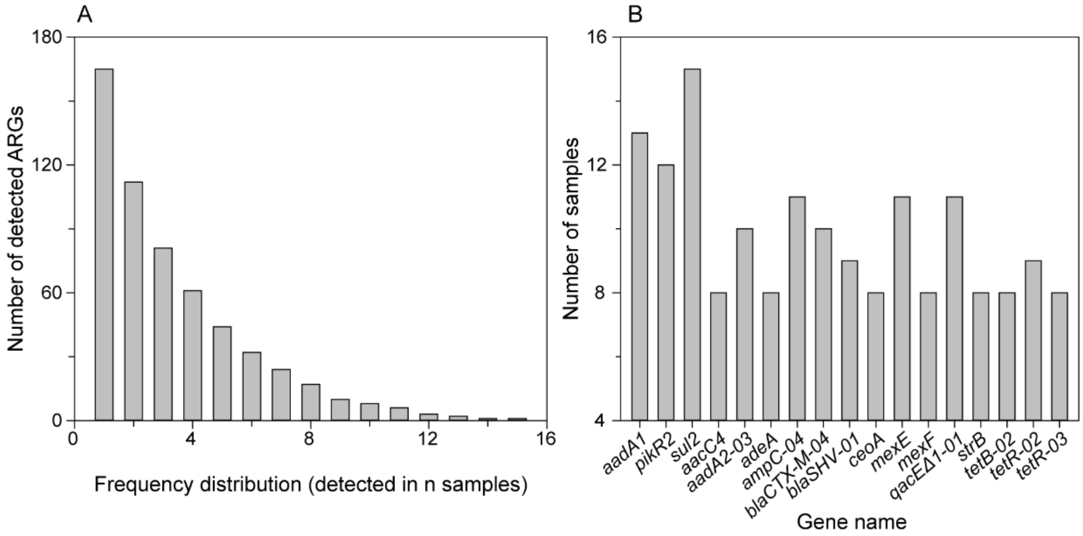

Fig. S4. ARGs in reservoir waters of southeast China. (**A**) Frequency distribution of ARGs in reservoirs. (**B**) Number of samples in which ARGs can be detected, only prevalent ARGs (core and common ARGs) are shown.

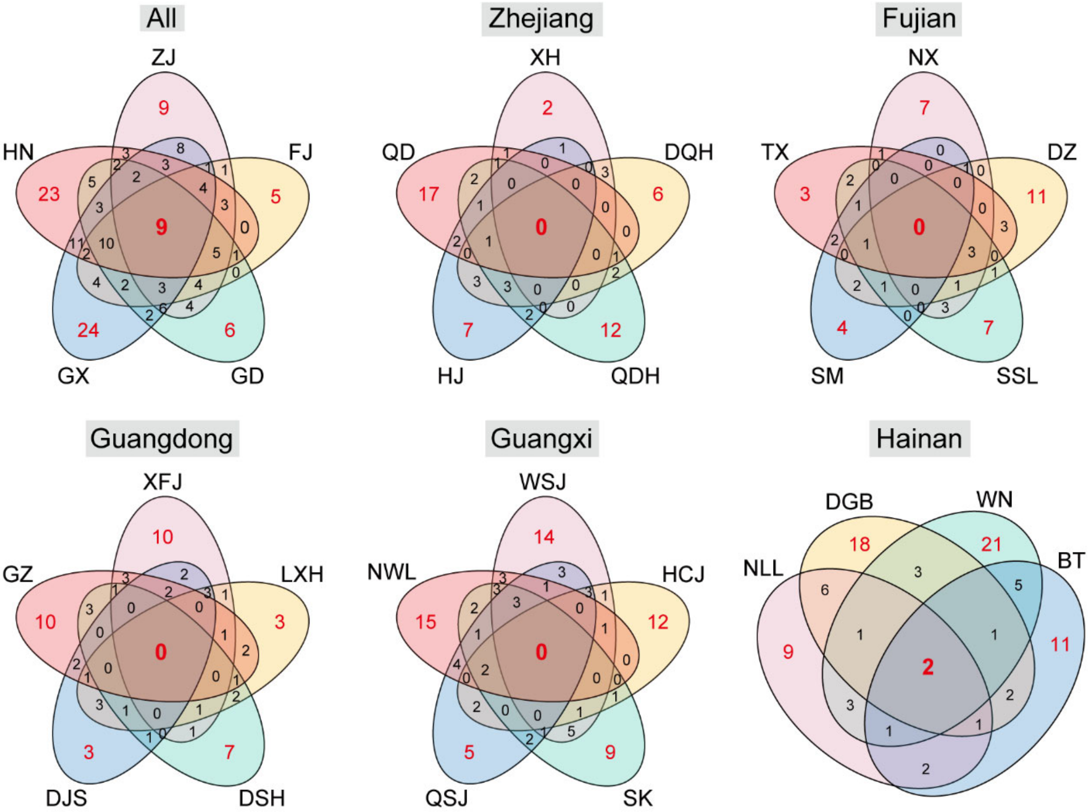

Fig. S5. The number of shared and unique ARGs among different reservoirs. All: all five provinces including Zhejiang, Fujian, Guangdong, Guangxi, and Hainan provinces. For detailed abbreviation information of each reservoir see Table S1.

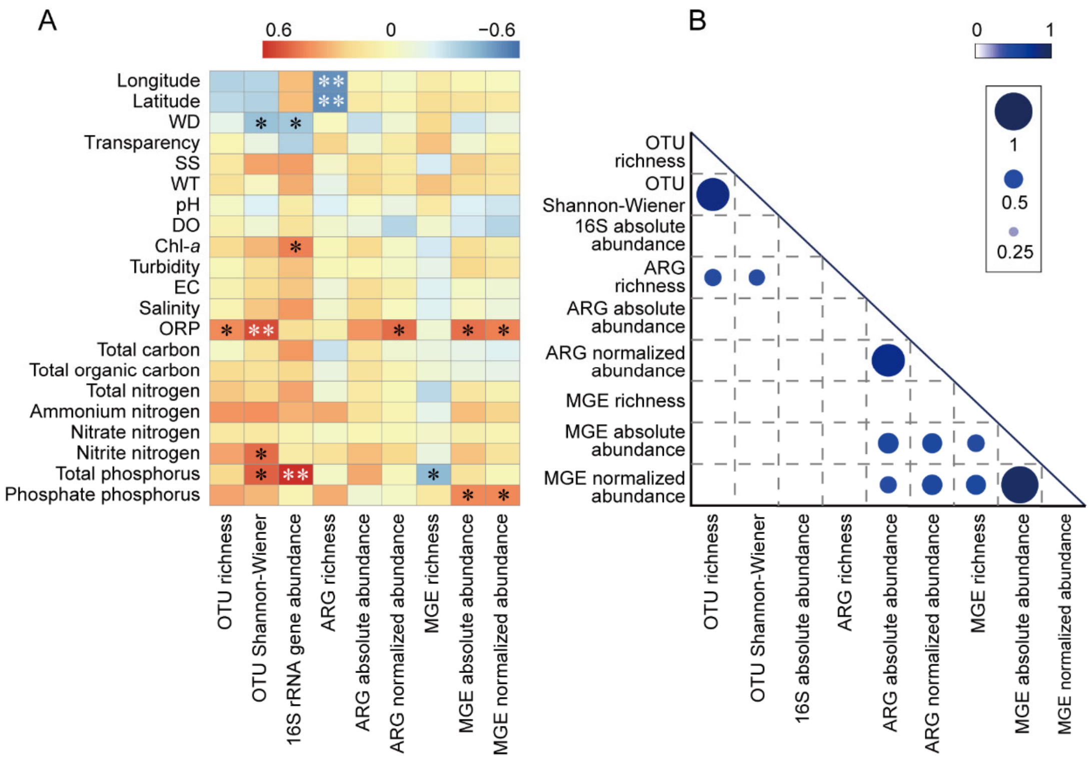

Fig. S6. Spearman's correlation between diversity and abundance of bacterial communities, ARGs, MGEs and geographical or environmental factors. (**A**) Spearman's correlation between environmental factors and bacterial communities, ARGs, and MGEs. * $P < 0.05$, ** $P < 0.01$. (**B**) Spearman's correlation between bacterial OTUs, ARGs and MGEs. 16S represents 16S rRNA gene. Note that only significant relationships are showed in the diagram. WD, water depth; SS, suspended solid; WT, water temperature; DO, dissolved oxygen; EC, electrical conductivity; ORP, oxidation-reduction potential.

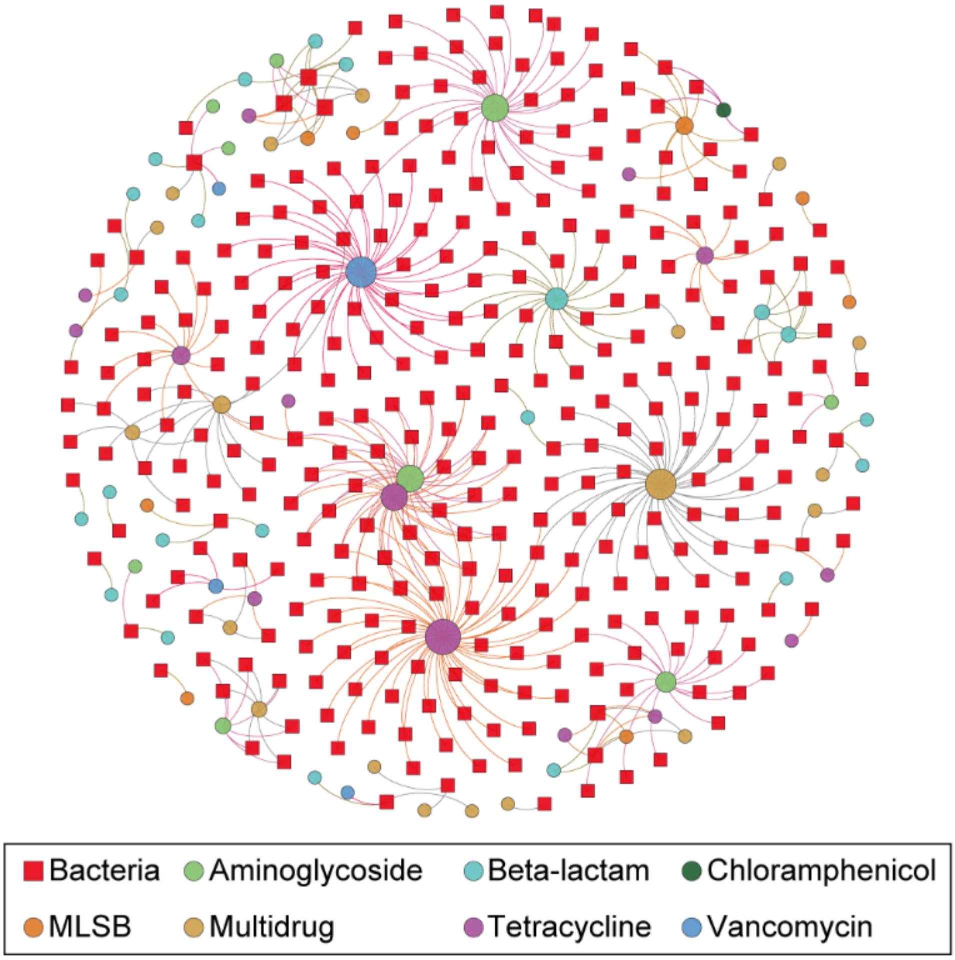

Fig. S7. Network analysis showing significant correlations between bacterial OTUs and ARGs. The size of node corresponds to the number of connections, and the color of the node was assigned according to different ARG classes.

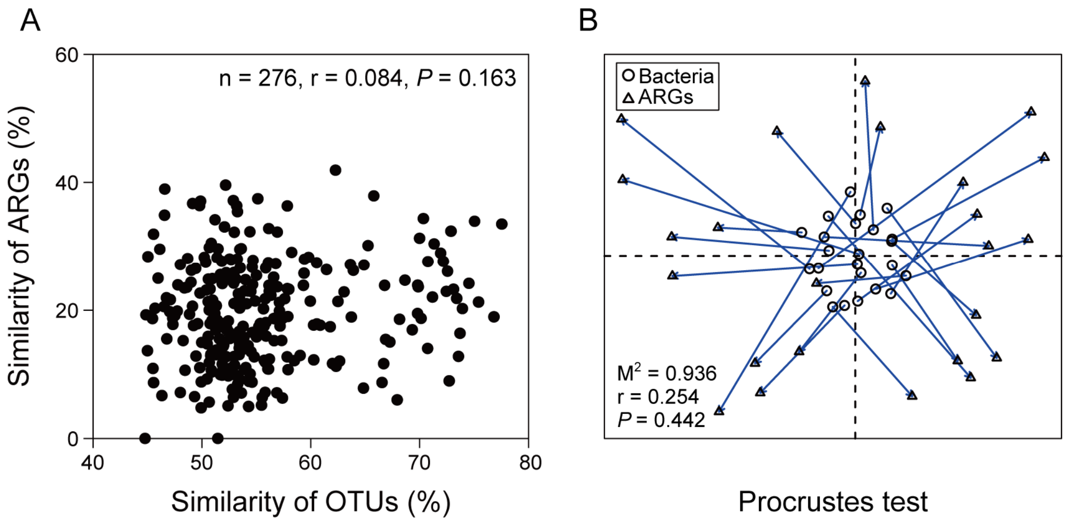

Fig. S8. Relationship between bacterial communities and ARG profiles. (**A**) Spearman's correlation between the Bray-Curtis similarity of bacterial communities and ARG profiles. (**B**) Procrustes test showing the correlation between ARG profiles and bacterial communities based on Bray-Curtis similarity metrics (M^2 = 0.936, r = 0.254, *P* = 0.442, 999 permutation).

Table S1. Information of 24 sampling reservoirs in this study.

Reservoir name	Sample ID	Region	Lat. (°N)	Long. (°E)	Province
Xihu	XH	ZJ	30.2391	120.1426	Zhejiang
Dongqianhu	DQH	ZJ	29.7679	121.6629	Zhejiang
Xin’anjiang	QDH	ZJ	29.5359	118.9782	Zhejiang
Henjin	HJ	ZJ	29.2352	120.4731	Zhejiang
Qiaodun	QD	ZJ	27.4754	120.3054	Zhejiang
Nanxi	NX	FJ	27.3428	120.1222	Fujian
Dongzhen	DZ	FJ	25.4843	118.9453	Fujian
Sanshiliujiaohu	SSL	FJ	25.4786	119.7636	Fujian
Shanmei	SM	FJ	25.1960	118.4288	Fujian
Tingxi	TX	FJ	24.8033	118.1394	Fujian
Xinfengjiang	XFJ	GD	23.7923	114.5865	Guangdong
Liuxihe	LXH	GD	23.7575	113.7882	Guangdong
Dashahe	DSH	GD	22.5447	112.3987	Guangdong
Dajingshan	DJS	GD	22.2977	113.5488	Guangdong
Gaozhou	GZ	GD	22.0502	111.0419	Guangdong
Wangshengjiang	WSJ	GX	21.9051	109.6093	Guangxi
Hongchaojiang	HCJ	GX	21.8219	109.1519	Guangxi
Shikang	SK	GX	21.7591	109.3507	Guangxi
Qingshuijiang	QSJ	GX	21.6964	109.2698	Guangxi
Niuweiling	NWL	GX	21.5973	109.2382	Guangxi
Niululing	NLL	HN	18.9863	110.1488	Hainan
Daguangba	DGB	HN	18.9802	109.0062	Hainan
Wanning	WN	HN	18.7822	110.3127	Hainan
Beitou	BT	HN	18.7233	110.2690	Hainan

ZJ: Zhejiang province; FJ: Fujian province; GD: Guangdong province; GX: Guangxi province; HN: Hainan province.

Table S2. The total absolute and normalized abundance of bacteria, ARG and MGE in each reservoir.

Reservoir	16S rRNA gene abundance (copies/L)	ARG absolute abundance (copies/L)	ARG normalized abundance	MGE absolute abundance (copies/L)	MGE normalized abundance
Xihu	7.65E+09	1.12E+08	1.46E-02	9.47E+07	1.24E-02
Dongqianhu	1.01E+10	4.47E+08	4.43E-02	2.61E+08	2.59E-02
Xin'anjiang	3.38E+09	4.34E+07	1.28E-02	1.28E+07	3.80E-03
Henjin	1.62E+09	3.96E+07	2.45E-02	8.73E+06	5.40E-03
Qiaodun	4.31E+09	2.95E+07	6.84E-03	5.62E+07	1.30E-02
Nanxi	2.29E+09	3.08E+07	1.34E-02	4.10E+06	1.79E-03
Dongzhen	3.56E+09	2.40E+07	6.75E-03	1.36E+06	3.82E-04
Sanshiliujiaohu	3.02E+09	2.18E+07	7.21E-03	2.38E+06	7.88E-04
Shanmei	4.97E+09	7.38E+06	1.48E-03	0.00E+00	0.00E+00
Tingxi	3.98E+09	4.71E+06	1.18E-03	1.82E+07	4.58E-03
Xinfengjiang	6.07E+08	2.22E+06	3.66E-03	3.94E+06	6.49E-03
Liuxihe	1.34E+09	2.01E+07	1.49E-02	3.63E+06	2.70E-03
Dashahe	3.15E+09	4.79E+07	1.52E-02	1.26E+07	4.01E-03
Dajingshan	2.84E+09	5.31E+06	1.87E-03	3.88E+06	1.37E-03
Gaozhou	3.58E+09	5.06E+07	1.41E-02	3.56E+05	9.93E-05
Wangshengjiang	2.53E+09	1.51E+07	5.96E-03	1.28E+07	5.07E-03
Hongchaojiang	1.53E+09	2.43E+07	1.59E-02	2.06E+07	1.35E-02
Shikang	3.10E+09	1.45E+08	4.67E-02	1.96E+07	6.31E-03
Qingshuijiang	3.06E+09	1.62E+07	5.29E-03	0.00E+00	0.00E+00
Niuweiling	4.30E+09	5.65E+07	1.31E-02	1.31E+08	3.05E-02
Niululing	1.88E+09	4.65E+07	2.48E-02	1.42E+07	7.56E-03
Daguangba	2.90E+09	4.71E+07	1.62E-02	3.02E+07	1.04E-02
Wanning	1.60E+09	1.54E+07	9.60E-03	4.91E+06	3.07E-03
Beitou	6.61E+09	2.97E+07	4.49E-03	2.32E+06	3.51E-04
Mean ± SE	3.50E+09 ± 4.36E+08	5.34E+07 ± 1.84E+07	1.35E-02 ± 2.41E-03	3.00E+07 ± 1.20E+07	6.65E-03 ± 1.61E-03

Note that the normalized abundance is the ratio of ARGs and MGEs to 16S rRNA gene.

Table S3. Spearman's correlation between bacterial taxonomy and resistant function at phylum and ARG class levels, respectively.

	Aminoglycoside	Beta-lactam	Chloramphenicol	MLSB	Multidrug	Others	Sulfonamide	Tetracycline	Vancomycin	MGEs
Acidobacteria	0.011	0.050	0.037	–0.090	0.035	–0.003	0.015	–0.056	–0.021	–0.010
Actinobacteria	0.088	0.047	0.066	**0.149***	0.036	0.016	–0.003	0.099	–0.020	0.111
Bacteroidetes	0.151	–0.053	0.097	–0.029	–0.030	–0.090	–0.138	0.013	–0.183	0.015
Chloroflexi	**0.201***	0.023	–0.010	–0.115	–0.101	–0.193	–0.084	0.095	–0.225	0.138
Cyanobacteria	–0.007	**0.166***	0.040	–0.089	–0.006	–0.163	–0.202	**0.150***	–0.113	0.018
Firmicutes	–0.112	–0.052	0.048	0.145	0.034	0.053	–0.068	0.115	–0.086	0.086
Patescibacteria	0.133	–0.007	0.139	0.009	0.010	0.127	0.012	**0.150***	0.061	–0.040
Planctomycetes	0.149	0.014	0.087	0.010	–0.078	–0.233	–0.083	0.087	–0.120	–0.027
Proteobacteria	0.078	0.003	**0.154***	0.090	0.029	–0.133	–0.078	0.078	–0.012	0.069
Verrucomicrobia	0.079	0.074	–0.029	–0.214	–0.084	–0.288	–0.072	–0.107	–0.084	0.013

MLSB, macrolide-lincosamide-streptogramin B.

Significant correlations ($P < 0.05$) are shown in bold.

* $P < 0.05$; ** $P < 0.01$

Table S4. Spearman's correlation between ARG classes and eight MGE marker genes based on absolute abundance.

	Aminoglycoside	Beta-lactam	Chloramphenicol	MLSB	Multidrug	Others	Sulfonamide	Tetracycline	Vancomycin
intI-1	0.077	–0.224	–0.218	–0.250	**0.427***	–0.049	0.151	–0.090	–0.030
IS613	–0.015	0.136	0.137	–0.075	0.015	0.084	–0.046	–0.015	–0.259
tnpA-01	–0.006	0.129	0.304	–0.004	–0.328	0.163	–0.063	0.085	0.069
tnpA-02	0.095	**0.495***	–0.272	**0.431***	0.339	–0.107	0.102	0.253	0.263
tnpA-03	0.015	0.015	0.198	0.075	0.196	0.387	0.198	0.015	–0.107
tnpA-04	–0.145	0.105	0.343	–0.085	–0.211	–0.129	**–0.520****	–0.123	0.185
tnpA-05	0.347	–0.020	0.129	0.159	0.237	0.198	0.038	0.246	–0.134
Tp614	–0.366	**–0.578****	–0.253	**–0.421***	0.078	0.146	0.026	–0.329	0.094

The data used were **absolute** abundance (copy number per litre water).

Further information regarding the PCR primers can be found in Guo et al. 2018 (ref. 10).

MLSB, macrolide-lincosamide-streptogramin B.

* $P < 0.05$, ** $P < 0.01$.

Table S5. Spearman's correlation between ARG classes and eight MGE marker genes based on normalized abundance.

	Aminoglycoside	Beta-lactam	Chloramphenicol	MLSB	Multidrug	Others	Sulfonamide	Tetracycline	Vancomycin
intI-1	0.051	–0.284	–0.276	–0.323	0.314	–0.057	0.162	–0.068	0.044
IS613	0.045	0.196	0.229	0.015	0.075	0.084	0.046	0.106	–0.259
tnpA-01	0.037	0.098	0.341	–0.015	–0.135	0.218	0.035	0.138	0.129
tnpA-02	0.012	**0.491***	–0.231	0.306	0.260	–0.086	0.012	0.185	0.221
tnpA-03	–0.015	0.045	0.168	0.196	0.136	0.387	0.198	0.015	–0.137
tnpA-04	–0.105	0.096	0.311	–0.055	–0.191	–0.097	–0.354	–0.012	0.350
tnpA-05	0.362	–0.080	0.069	0.243	0.149	0.209	0.062	0.229	–0.153
Tp614	–0.365	**–0.567****	–0.283	**–0.419***	0.227	0.179	0.009	–0.346	0.120

The data used were **normalized** abundance (ARGs and MGEs absolute abundance/16S rRNA gene absolute abundance).

Further information regarding the PCR primers can be found in Guo et al. 2018 (ref. 10).

MLSB, macrolide-lincosamide-streptogramin B.

* $P < 0.05$, ** $P < 0.01$.

Table S6. The co-occurrence patterns between ARGs and MGE marker genes.

MGEs	Co-occurring ARGs	Co-occurring ARGs class	Spearman's correlation coefficient
IS613	*aadA2-02*	Aminoglycoside	0.72
	aadA5-02	Aminoglycoside	1.00
	blaPAO	Beta-lactam	0.60
	oprD	Beta-lactam	0.66
	erm(36)	MLSB	0.66
	oprJ	Multidrug	0.66
	putitive multidrug	Multidrug	1.00
	nimE	Others	0.66
	tet(34)	Tetracycline	0.60
tnpA-01	*blaCTX-M-01*	Beta-lactam	0.67
tnpA-02	*blaSHV-01*	Beta-lactam	0.68
	tetE	Tetracycline	0.64
	tetL-02	Tetracycline	0.70
tnpA-03	*aacA/aphD*	Aminoglycoside	0.66
	aacC1	Aminoglycoside	0.66
	aadA9-01	Aminoglycoside	1.00
	aadD	Aminoglycoside	0.72
	spcN-01	Aminoglycoside	1.00
	ampC-06	Beta-lactam	0.72
	blaCMY2-01	Beta-lactam	1.00
	imiR	Beta-lactam	1.00
	pikR1	MLSB	0.66
	mtrC-01	Multidrug	0.66
	qacA/qacB	Multidrug	1.00
	sul1	Sulfonamide	0.72
	vanSE	Vancomycin	1.00
tnpA-04	*acrR-01*	Multidrug	0.60
tnpA-05	*aacC*	Aminoglycoside	0.74
Tp614	*vanC2/vanC3*	Vancomycin	0.71

Further information regarding the PCR primers can be found in Guo et al. 2018 (ref. 10).

MLSB, macrolide-lincosamide-streptogramin B.

Only connections with a strong (Spearman's correlation coefficient $r \geq 0.6$) and significant ($P < 0.01$) correlation are presented.
